# Non‐coding RNAs in aortic dissection: From biomarkers to therapeutic targets

**DOI:** 10.1111/jcmm.15802

**Published:** 2020-09-04

**Authors:** Mengdie Cheng, Yanyan Yang, Hai Xin, Min Li, Tingyu Zong, Xingqiang He, Tao Yu, Hui Xin

**Affiliations:** ^1^ Department of Cardiology The Affiliated hospital of Qingdao University Qingdao China; ^2^ Department of Immunology School of Basic Medicine Qingdao University Qingdao China; ^3^ Department of Vascular Surgery The Affiliated Hospital of Qingdao University Qingdao China; ^4^ Institute for Translational Medicine The Affiliated Hospital of Qingdao University Qingdao China; ^5^ Department of Cardiac Ultrasound The Affiliated Hospital of Qingdao University Qingdao China

**Keywords:** aortic dissection, non‐coding RNAs, potential biomarkers, therapeutic targets

## Abstract

Aortic dissection (AD) is the rupture of the aortic intima, causing the blood in the cavity to enter the middle of the arterial wall. Without urgent and proper treatment, the mortality rate increases to 50% within 48 hours. Most patients present with acute onset of symptoms, including sudden severe pain and complex and variable clinical manifestations, which can be easily misdiagnosed. Despite this, the molecular mechanisms underlying AD are still unknown. Recently, non‐coding RNAs have emerged as novel regulators of gene expression. Previous studies have proven that ncRNAs can regulate several cardiovascular diseases; therefore, their potential as clinical biomarkers and novel therapeutic targets for AD has aroused widespread interest. To date, several studies have reported that microRNAs are crucially involved in AD progression. Additionally, several long non‐coding RNAs and circular RNAs have been found to be differentially expressed in AD samples, suggesting their potential roles in vascular physiology and disease. In this review, we discuss the functions of ncRNAs in AD pathophysiology and highlight their potential as biomarkers and therapeutic targets for AD. Meanwhile, we present the animal models previously used for AD research, as well as the specific methods for constructing mouse or rat AD models.

## 1 INTRODUCTION

**FIGURE 1:**
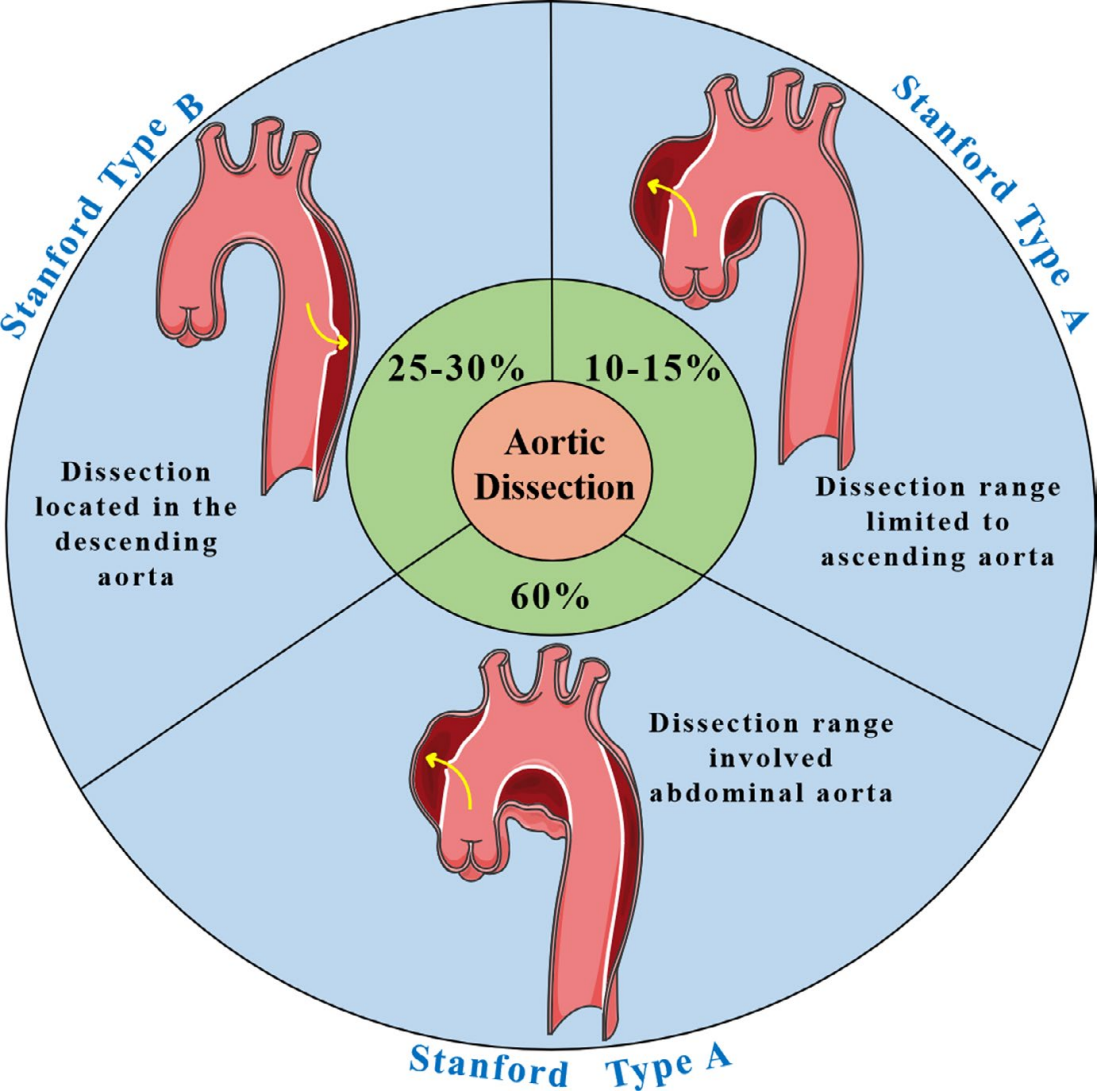
The Stanford classification of aortic dissection. There are mainly two types: Stanford A with dissection range limited to ascending aorta or dissection range involved abdominal aorta, and Stanford B with dissection located in the descending aorta

Aortic dissection (AD) is a fatal vascular disease defined as the intimal tear of the aorta, which causes the blood in the cavity to pass into the middle of the artery wall, and eventually forms the dissected haematoma and the true and false lumen expanding along the long axis of the artery.^1^ Accumulating evidence indicates that the annual incidence of AD is 4.586 per 100 000 persons aged 65‐75 years, with more than 35 deaths per 100 000 persons.^2^ If left untreated, about 24% of the patients die within the first 24 hours and 50% die within 48 hours.^3^ In short, AD is an acute and severe disease with a high mortality rate. There are two classification systems for AD: Stanford, which is the most commonly used, and DeBakey. The Stanford classification is further divided into two types—A and B (Figure 1). Stanford type A AD originates from the ascending aorta and is divided into two subtypes according to the involvement of the abdominal aorta or the limitation to the ascending aorta. It is usually treated by sternotomy, especially for emergency surgical intervention, whereas Stanford type B AD does not involve the ascending aorta and is usually treated using drugs, unless the condition is complex.^4^ AD can also be categorized as acute, subacute and chronic, specifically at two time‐points (2 weeks and 3 months).^3^

Studies have demonstrated that several factors can influence AD. For example, various genetic mutations were reported to lead to the occurrence and progression of ADs, such as Marfan syndrome (*FBN1* mutations),^5^ Ehlers‐Danlos syndrome (*COL3A1* mutations)^6^ and Loeys‐Dietz syndrome (*TGFBR1* or *TGFBR2* mutations).^7^ Moreover, infections and vascular inflammation, which involves some inflammatory factors including matrix metalloproteinases (MMPs) and vascular endothelial growth factor (VEGF), and vascular smooth muscle cell (VSMC) phenotypic transition are also significantly involved in AD. Additionally, bicuspid aortic valve, one of the most common cardiovascular malformations with an incidence of 1%‐2% in the general population, is also reported to be closely related to AD development^8^ mainly owing to lack of elastic fibre components and increased release of matrix metalloproteinases.^9^ Recently, increased studies focus on the participation of non‐coding RNAs (ncRNAs) in various diseases.^10^, ^11^, ^12^, ^13^ ncRNAs are mainly divided into two main categories according to their length: small ncRNA and long ncRNA. They normally cannot encode proteins, which were regarded as ‘junk’ transcriptional products. However, recent studies have found that ncRNAs are functional regulatory molecules that can regulate gene expression at the transcription and post‐transcription levels. Particularly, numerous studies have reported that non‐coding RNAs (ncRNAs) play a significant role in the development of cardiovascular diseases, such as atherosclerosis, restenosis after stent and aneurysm.^14^, ^15^, ^16^, ^17^, ^18^ Likewise, several reports have suggested that ncRNAs also play a crucial regulatory role in the occurrence and development of AD.^19^, ^20^, ^21^ In this review, we discuss the role of ncRNAs and their target genes, as well as their regulatory mechanisms in AD. In this review, we highlight some previously identified ncRNAs that are involved in multiple processes that lead to AD. In‐depth research of these ncRNAs can provide new insights into the treatment and prevention of AD and serve as potentially effective biomarkers and therapeutic targets for predicting the risk of AD and evaluating prognosis in clinical settings. Furthermore, smoking, hypertension, atherosclerosis, hyperlipidaemia and age are certain risk factors that contribute to the occurrence of AD.^22^

## 2 THE PATHOGENESIS OF AD

The pathogenesis of AD includes vascular inflammation, activity of MMPs and phenotype switching of VSMCs.^23^ Damage to the endothelial cells (ECs) triggers vascular inflammation, which is regulated by the immune response mechanism, and it subsequently activates MMP, an enzyme used to degrade the extracellular matrix (ECM). The imbalance between MMPs and TIMPs directly contributes to the remodelling of the aortic wall, which is a central link in the formation of AD.^24^ It has also been reported that the macrophages and their related products localized within the walls of the aorta can trigger and maintain the thoracic aortic dissection (TAD) inflammation and matrix degradation.^25^ Collagen synthesis and MMP‐2 (gelatinase A) production are elevated in the synthetic phenotype of VSMCs and can promote collagen deposition and type IV collagen and elastin degradation.^26^ These evidences suggested that AD is a very complex process involving inflammation and matrix degradation.

Media degradation is a major histopathological feature of AD, which includes the severe degradation of the ECM that is associated with the depletion of the smooth muscle cells (SMCs), rupture of elastic fibres and collagen degradation, consequently weakening the artery wall and eventually leading to the formation of AD.^27^ The VSMCs in the middle layer of the aorta play an important role in maintaining aortic wall homeostasis. Notably, dysfunction in the proliferation and migration of VSMCs has been reported to be associated with vascular diseases.^28^ Therefore, VSMCs are also believed to be responsible for the formation of AD. During AD pathogenesis, the dysfunctional VSMCs are considered to be one of the most important factors, mainly including apoptosis and phenotype switching.^29^ VSMC phenotypic transformation is a biological process which is characterized by the transformation of a contractile (differentiated) phenotype into a synthetic (dedifferentiated) phenotype that could occur under the influence of the environment including signal transduction, gene transcription and epigenetic modification.^30^ Several molecular mechanisms are involved in this process, such as serum response factor (SRF), Krüppel‐like factor 4 (KLF4), forkhead box O 4 (FOXO4), microRNAs, ten‐eleven‐translocation 2 (TET2), Rho‐actin and transforming growth factor‐β (TGF‐β).^31^ Two VSMC‐derived ECM proteins, collagen and elastin, have been implicated with apoptosis and phenotype switching during AD pathogenesis. Apoptosis of VSMCs and elastin fibre fragmentation can lead to the degeneration of the ECM, thereby weakening the strength and elasticity of the vessel wall,^9^ whereas abnormal phenotypic switching can promote collagen deposition and elastin degradation.^26^ These two processes directly influence the development of AD; however, their underlying molecular mechanisms remain to be elucidated. Different stimuli, such as growth factors, chemical factors, cell adhesion molecules, ECM enzymes and damage‐stimulating signals, can also result in the occurrence of AD.^32^

Hypertension has also been confirmed as a risk factor that contributes to the occurrence of AD, which represents an important haemodynamic basis.^9^ For example, miR‐21 has been shown to be involved in the development of hypertension and target organ damage (TOD) by regulating renin‐angiotensin‐aldosterone system (RAAS) and peripheral blood mononuclear cells.^33^ miR‐505 promotes the development of hypertension by affecting the function of endothelial cells.^34^ Particularly, vascular endothelial cells and haemodynamics were reported to be significant for vascular homeostasis and hypertension.^35^ Shear stress is a force per unit area, which is generated when tangential force (blood flow) acts on the inner membrane of vessel (endothelial cells), and plays an important role in determining the pathological origin of arterial disease.^36^ In addition, shear stress is also associated with endothelial phenotype changes, which are related to atherosclerosis.^37^ Recently, a large number of literature reported that a series of microRNAs can regulate haemodynamics and endothelial cell behaviour, thus participating in the occurrence of aortic diseases. microRNA‐126 was shown to be involved in the signal transmission from endothelial cells to SMC, thereby inducing SMC turnover which was affected by haemodynamic shear stress.^38^ It was also reported that Krüppel‐like factor 2 (KLF2) can regulate the gene expression pattern of endothelial cells under the stimulation of atheroprotective flow. KLF2 transduction or shear stress stimulation can induce endothelial cells to secrete extracellular vesicles to enrich miR‐143/145 and control the target gene expression of co‐cultured SMC, which is of great significance in combating atherosclerosis.^39^ Moreover, haemodynamic changes and endothelial cell dysfunction are also crucially involved in the pathogenesis of AD. Recent reports demonstrated that EC dysfunction participates in the development of AD through endoplasmic reticulum (ER) stress depending on microparticles derived from SMCs, and eventually leads to EC apoptosis and inflammation.^40^ The modification of protein S‐nitrosylation (SNO) in endothelial cells can result in the destruction of the endothelial barrier, which ultimately leads to the formation of TAD.^41^ Currently, some studies revealed the regulatory roles of microRNAs on endothelial cells behaviour during aortic dissection. For example, miR‐27a promotes the activation of apoptotic pathway by targeting the expression of fas‐associated protein with death domain (FADD), thereby promoting the apoptosis of EC, whereas the treatment of miR‐27a activator targeting ECs apoptosis can significantly reduce the incidence of AD.^42^ Together, these evidences provide us with new insights into the occurrence and development of AD, and suggest more comprehensive prevention and diagnostic methods.

## 3 CLINICAL EVALUATION OF MIRNAS IN AD PATIENTS

As mentioned above, AD is an acute and severe disease with a high mortality rate. The annual incidence of AD is 4.586 per 100 000 persons aged 65‐75 years, with more than 35 deaths per 100 000 persons.^2^ Without urgent and proper treatment, about 24% of the patients die within the first 24 hours and 50% die within 48 hours.^3^ Therefore, early diagnosis of AD is particularly important for reducing mortality and improving prognosis. Given their significant regulatory roles, ncRNAs, especially miRNAs, have already been used for clinical testing.^43^ Several ncRNAs have been identified as critical regulators of AD; hence, we reviewed the pathophysiology of AD regulated by ncRNAs including miRNAs (Table 1), lncRNAs (Table 2) and circRNAs (Table 3), and the diagnostic and therapeutic potential of some statistically different ncRNAs detected in the tissue or blood samples of AD patients and healthy people. We also summarized the information regarding the identified ncRNAs and their diagnostic values, particularly their sensitivity and specificity, and the four miRNAs (miR‐25, miR‐29a, miR‐155 and miR‐26b) showed more reliable diagnostic value with a sensitivity of 92%, a specificity of 93.33% and area under curves (AUC) of 0.973 ^44^, ^45^ (Table 4). And we listed novel potential biomarkers that need to be further validated in Table 5.^46^, ^47^, ^48^, ^49^, ^50^ The expression of these non‐coding RNAs in the peripheral blood or aortic tissues of AD patients and healthy people was obviously different (fold change > 2), which has potential clinical significance as early diagnostic biomarkers. However, their regulatory function and underlying mechanisms in the occurrence and development of AD need to be further explored.

**Table 1:** Representative miRNAs regulating pathophysiology of AD

miRNA	Sample type	Cell	Regulation	Target	Function	Mechanism	Ref.
miR‐21	NA	VSMC	Increased	SMAD7	Promote phenotypic transition	TGF‐β signalling pathway	^63^
miR‐134‐5p	Thoracic aorta	VSMC	Decreased	STAT5B/ITGB1	Inhibit phenotypic transition/apoptosis	ITGB1/CRE signalling pathway	^19^
miR‐145	Ascending aorta	VSMC	Decreased	CTGF	Inhibit apoptosis	TGF‐β/SMAD3 signalling pathway	20, 67
miR‐320	Peripheral blood	Monocytes, macrophage	Decreased	MMP2/9	Inhibit degradation/remodelling of the ECM	Post‐transcriptional control	^69^
miR‐320d/582	Thoracic aorta	VSMC	Decreased	TRIAP1/NET1, COLIA1/SPP1	Inhibit apoptosis	Apoptotic pathway	^21^
miR‐144‐3p	Dissection specimens	VSMC	Increased	TE	Reduce elastin	Protein translation	^73^
miR‐146b	Peripheral blood	VSMC, EC, MPh	Increased	NF‐κB1/TRAF6/MMP6/ACTA2	Promote inflammation/apoptosis/ECM degradation	TLR/TGF‐β signalling pathway	^76^
miR‐146a‐5p	TAAD plasma sample and tissue	VSMC	Increased	SMAD4	Promote the proliferation and migration of VSMC	TGF‐β signalling pathway	^82^
miR‐30a	Ascending aorta	VSMC	Increased	LOX	Inhibit cross‐link collagen and elastin	Inhibit the protein abundance of LOX	^83^
miR‐143/145	Ascending aorta	VSMC	Decreased	TGF‐β1/P38‐MAPK	Inhibit phenotypic transition	TGF‐β1 signalling pathway	12, 52
miR‐4787‐5p/4306	Peripheral blood	VSMC	Increased	PKD1/TGF‐β1	Damage cell‐cell adhesion/inflammation	TGF‐β1 signalling pathway	^90^
miR‐26b	Ascending aorta	VSMC	Increased	HMGA2	Promote apoptosis EMT	TGF‐β signalling pathway	^94^

Abbreviations: EC, endothelial cell; ECM, extracellular matrix; EMT, endothelial‐mesenchymal transition; MPh, macrophages; TAAD, thoracic aortic aneurysm and dissection; TE, tropoelastin; VSMC, vascular smooth muscle cell.

**Table 2:** Summary of lncRNAs regulating pathophysiology of AD

lncRNA	Sample type	Cell	Regulation	Target	Function	Potential biomarker or target	Ref.
lncP2RX7	TAD ascending aorta	VSMC	Increased	P2RX7	Promote inflammation	Yes	^113^
HIF1A‐AS2		VSMC	Increased	HIF1A	Inhibit the proliferation and migration of AoSMCs	Yes	
AX746823		VSMC	Increased	RUNX1	Promote inflammation	Yes	
RP11‐69I8.3		VSMC	Increased	CTGF	Promote apoptosis	Yes	
RP11‐536K7.5		VSMC	Increased	IL2RA	Promote inflammation	Yes	
CDKN2B‐AS1		VSMC	Increased	CDKN2B	Promote apoptosis	Yes	
ENSG00000269936	TAD ascending aorta	VSMC	Increased	MAP2K6	Inhibit collagen synthesis	Yes	^97^
lncRNA‐1421		VSMC	Decreased	ACTA2/FBLN5/TIMP3	Decrease SMC contraction/inhibit VSMC proliferation and migration.	Yes	
lncRNA‐XIST		VSMC	Increased	P21	Inhibit VSMC proliferation	Yes	
ENSG00000248508		VSMC	Increased	Up‐regulated by RUNX1	Promote inflammation	Yes	
ENSG00000226530		VSMC	Increased		Promote inflammation	Yes	
EG00000259719		VSMC	Increased		Promote inflammation	Yes	
PTENP1	AD tissues and adjacent aortic tissue specimens	VSMC	Increased	miR‐21	Promote apoptosis	Yes	^107^

Abbreviations: AoSMCs, aortic smooth muscle cells; TAD, thoracic aortic dissectionVSMC, vascular smooth muscle cell.

**Table 3:** Summary of circRNAs regulating pathophysiology of AD

circRNAs	Sample type	Cell	Regulation	Target	Function	Potential diagnostic biomarker	Ref.
circMARK3	AAAD ascending aortic specimens	VSMC	Increased	miR‐1273	Promote inflammatory	Yes	^47^
circRNA‐101238	Type A TAD aortic specimens	VSMC	Increased	miR‐320a	Increase apoptosis	Yes	^119^
circRNA‐104634		VSMC	Increased	miR‐145‐3p	Promote phenotype switching	Yes	
circRNA‐104349		VSMC	Increased	miR‐26a‐3p	Increase apoptosis	Yes	
circRNA‐102683		VSMC	Decreased	miR‐29b‐1‐5p	Promote apoptosis/ECM degradation	Yes	
circRNA‐104033		VSMC	Decreased	miR‐195‐3p	Stimulate collagen remodelling	Yes	

Abbreviations: AAAD, Stanford type A aortic dissection; ECM, extracellular matrix; TAD, thoracic aortic dissection; VSMC, vascular smooth muscle cell.

**Table 4:** Representative miRNAs for diagnosing acute aortic dissection with risk score analysis

miRNA	Sample type	Result	Sensitivity	Specificity	AUC (95% CI)	Ref.
miR‐25	AAAD peripheral blood	Up‐regulated	92.00%	76.67%	0.881	^44^
miR‐29a		Up‐regulated	80.00%	93.33%	0.899	
miR‐155		Up‐regulated	84.00%	83.33%	0.863	
miR‐26b		Down‐regulated	88.00%	90.00%	0.911	
miR‐15a	Peripheral blood	Up‐regulated	75.7%	100%	0.855	^45^
miR‐23a		Up‐regulated	91.9%	85.7%	0.925	
let‐7b		Up‐regulated	79.4%	92.9%	0.887	
hcmv‐miR‐US33‐5p		Up‐regulated	73.5%	85.7%	0.815	

Abbreviations: AAAD, Stanford type A aortic dissection; AUC, area under curves; CI, confidence interval.

**Table 5:** Novel potential biomarkers for diagnosing acute aortic dissection in patients (fold change > 2, q‐value ≤ 0.05)

Up‐regulated ncRNAs	Sample type	Reference	Down‐regulated miRNAs	Sample type	Ref.
hsa‐miR‐93	TAD aorta segments	^48^	hsa‐miR‐1268	Aortic dissection tissue	^49^
hsa‐miR‐485‐3p			hsa‐miR‐939		
hsa‐miR‐146b‐5p	Aortic dissection tissue	^49^	miR‐29a	TAD ascending aorta segments	^46^
hsa‐miR‐19a			miR‐29c		
hsa‐miR‐505			miR‐30 family except miR‐30c		
miR‐518e	AAD peripheral blood	^50^	miR‐3682	AAD peripheral blood	^50^
miR‐16			miR‐3196		
miR‐451			miR‐3162		
miR‐663			miR‐3131		
circUBA2	AAAD ascending aorta	^47^	circCEP70	AAAD ascending aorta	^47^
circARHGAP26			circFAM120B		
circCHSY1					

Abbreviations: AAAD, Stanford type A aortic dissection; AAD: acute aortic dissection; TAD, thoracic aortic dissection.

We believe that these ncRNAs can be developed into reliable molecular targets for the clinical diagnosis and treatment of AD in the future. Although there are limitations and challenges in the clinical application of ncRNAs, such as off‐target metabolism and delivery, the examples that we presented in this review highlight their potential utilization in creating strategies for the treatment of AD in the future.

## 4 MIRNAS IN AORTIC DISSECTION

**FIGURE 2:**
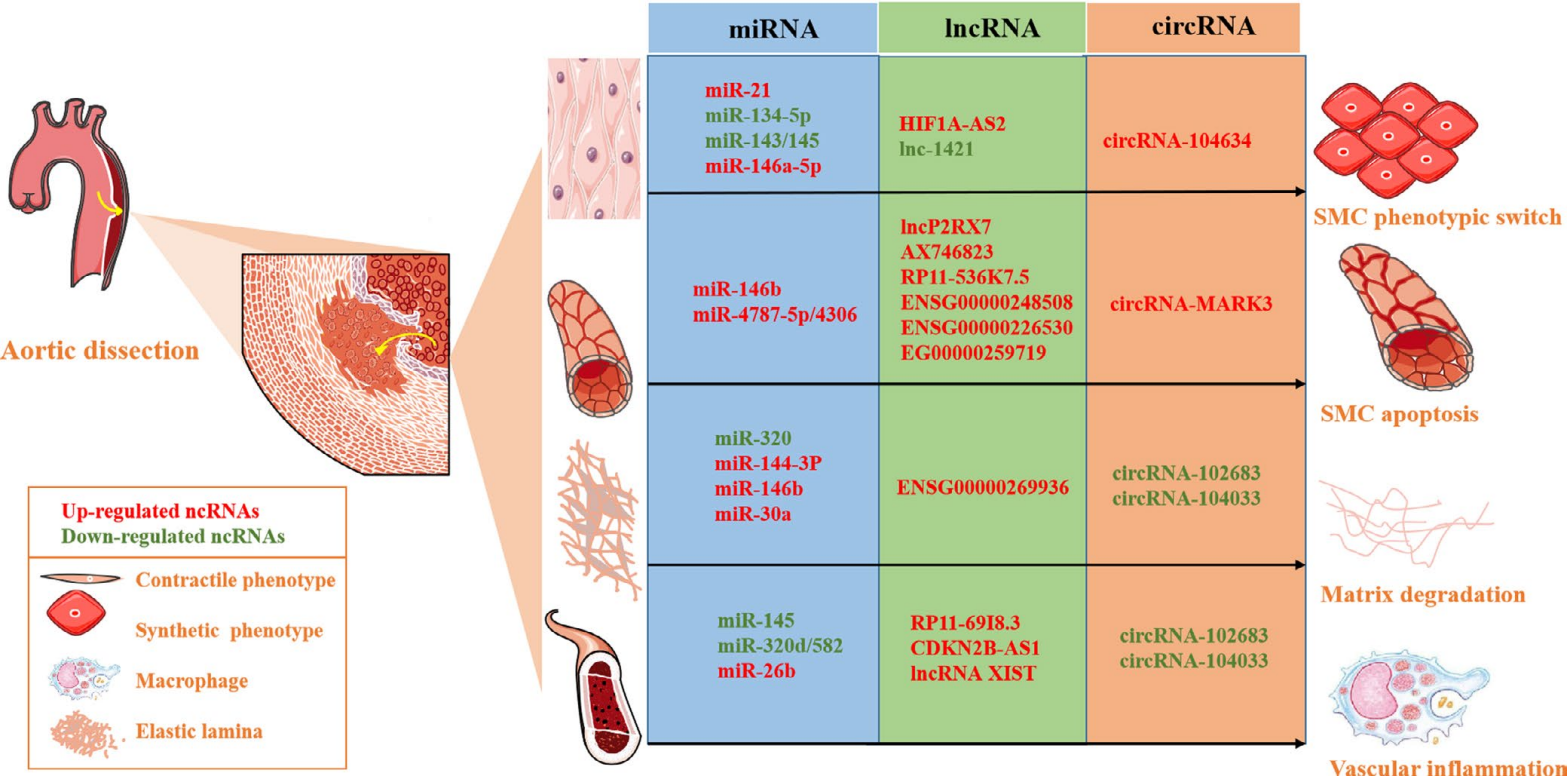
The function of non‐coding RNAs in aortic dissection. miRNAs, lncRNAs and circRNAs are all critically involved in biological regulation, including phenotypic transformation and apoptosis of VSMCs, matrix degradation and vascular inflammation

miRNAs are a class of endogenous ncRNAs approximately 20‐25 nucleotides (nt) long that play an essential part in modulating pathophysiological functions, such as differentiation, proliferation, migration and apoptosis. Mature miRNAs recognize the 3′‐untranslated region (3′‐UTR) of the target mRNA through base complementary pairing and either guide the degradation or repress translation of the target mRNA depending on degree of complementation. miRNAs are abundant and exist in a stable form in the plasma/serum.^51^ Changes in miRNA expression levels were observed in association with the occurrence of different diseases, such as miR‐183/96/182 cluster in retinal disorder,^10^ miR‐133b in neurodegenerative diseases ^11^ and miR‐143/145 in cardiovascular diseases ^52^; these changes can be detected by several methods, and the most common one is quantitative real‐time PCR (qRT‐PCR) assays.^53^ Therefore, we can diagnose certain diseases including cancers, such as pancreatic cancer,^54^ cholangiocarcinoma ^55^ and breast cancer,^56^ cardiovascular diseases including heart failure,^57^ acute myocardial infarction ^58^ and arrhythmias,^59^ by measuring the expression levels of circulating miRNAs, particularly, cardiovascular diseases, were significantly involved. Interestingly, under pathophysiological conditions, miRNAs have also been discovered to regulate VSMC phenotypic transition and consequently result in the development of AD.^60^ Here, we introduce several specific miRNAs that have been reported to play definite roles in the development of AD, and could be considered as potential diagnostic biomarkers therapeutic targets of AD (Figure 2) (Table 6).

**Table 6:** Representative animal models in AD studies

microRNA	Targets	Model	Sample type	Parameter measured	Ref.
miR‐21	SMAD7	S3^±^ mice infused with AngII	NA	Echocardiography, histological examination	^63^
miR‐134‐5p	STAT5B/ITGB1	Mice infused with AngII and high‐fat diet	TAD thoracic aorta	Ultrasonic histological examination	^19^
miR‐144‐3p	TE	BAPN solution dissolved in the drinking water	Dissection specimens	Blood pressure, histological examination	^73^
miR‐30a	Lysyl oxidase	Mice infused with AngII	AAD ascending aorta	Immunohistochemical staining	^83^

Abbreviations: AAD, acute aortic dissection; AngII, angiotensin II; BAPN, β‐aminopropionitrile monofumarate; TAD, thoracic aortic dissection; TE, tropoelastin.

### 4.1 miRNA

#### 4.1.1 miRNA‐21 (miR‐21)

VSMC phenotypic switching and ECM degradation directly affect AD.^23^ Phenotypic transformation from the contractile to the synthetic type can be induced by an abnormality in the TGF‐β signalling pathway, which further promotes the activation of the nuclear factor κ‐light‐chain‐enhancer of activated B cells (NF‐κB) and chemokine expression. Furthermore, the up‐regulation of the NF‐κB signalling pathway promotes chemokine expression, leading to VSMC inflammation ^61^ and ultimately facilitating the occurrence of AD. The expression of miR‐21 increased in a mouse AD model, which corroborates previously reported data in ascending thoracic aortic aneurysm (TAA) samples from patients.^62^ TAA is a pathological dilatation of the intima, media and adventitia of the aorta, although the final result of TAA expansion is to progress to TAAD, most TAA patients do not develop to TAAD, and the pathogenesis of TAA and TAAD is different. Moreover, miR‐21 knockout (miR‐21^−/−^) can aggravate the formation of SMAD3^±^ (mothers against decapentaplegic homolog 3) heterozygous (S3+/−) mice infused with AngII‐induced thoracic aortic aneurysm and dissection (TAAD) which occurs mainly in the ascending aorta.^63^ Mechanistically, miR‐21^−/−^ promoted SMAD7 expression and inhibited canonical TGF‐β signalling. SMAD7 overexpression further promoted the silencing of the TGF‐β signalling pathway in miR‐21^−/−^ mice, ultimately inducing the phenotypic transformation of VSMCs. At the same time, further experiments prove the lentiviral‐mediated silencing of *SMAD7* reversed the phenotype switching of VSMCs in S3^±^21^−/−^ mice. The proliferation and apoptosis rates of VSMCs, as well as the *MMP9* expression, were elevated in miR‐21^−/−^ mice. In contrast, collagen fibrillation was inhibited in miR‐21^−/−^ mice. Collectively, these results suggest that miR‐21 is a potential therapeutic target for TAAD.

#### 4.1.2 microRNA‐134‐5p (miR‐134‐5p)

miR‐134‐5p was previously implicated in the growth and migration of endothelial cells (ECs) and tuber formation during atherosclerosis.^64^ Recently, miR‐134‐5p was found to be highly down‐regulated in TAD thoracic aorta tissues via miRNA microarray screening assay. The overexpression of miR‐134‐5p in aortic smooth muscle cells (AoSMCs) significantly increased the expression of contractility markers and the formation of migration‐related stress fibres and significantly decreased the expression of ADAMTS‐1 and ADAMTS‐7 metalloproteinases. Furthermore, dual‐luciferase reporter assay confirmed that the signal transducer and activator of transcription 5B (*STAT5B*) and integrin beta‐1 (*ITGB1*) were direct downstream targets of miR‐134‐5p. *STAT5B*, which promotes growth factor expression and cell proliferation, is found to be activated during vascular injury, whereas *ITGB1* can inhibit the expression of VSMC contractile genes and the proliferation of AoSMCs.^65^, ^66^ Notably, miR‐134‐5p was also confirmed to inhibit tunica media degeneration and TAD evolution in vivo.^19^ In an AngII‐induced and high‐fat diet TAD model, Ad‐miR‐134‐5p significantly inhibited aortic dilatation and vascular media degeneration, thereby reducing the incidence of AD by 39%. These results suggest that the miR‐134‐5p, *STAT5B* and *ITGB1* in VSMCs may be potential therapeutic targets for AD.

#### 4.1.3 microRNA‐145 (miR‐145)

It has been proven that there is decreased miR‐145 expression in AAD ascending aorta samples.^52^ Subsequently, Huang et al confirmed that miR‐145 induces the proliferation, migration and apoptosis of VSMCs by targeting SMAD family member 3 (*SMAD3*) during AD.^67^ Another study reported that the miR‐145 expression level is consistent with the mortality of patients with AD. Increased miR‐145 expression can accelerate VSMC proliferation and ameliorate cell apoptosis by binding to *SMAD3* and connective tissue growth factor (CTGF), which can reduce the effect of miR‐145 in the progression of AAD.^20^ CTGF, a matricellular protein, can ameliorate aortic remodelling in the rat AD model and participate in the proliferation and apoptosis of VSMCs.^68^ In summary, studies on the miR‐145‐CTGF/SMAD3 axis may provide potential therapeutic targets for AAD.

#### 4.1.4 microRNA‐320 (miR‐320), microRNA‐320d (miR‐320d) and microRNA‐582 (miR‐582)

The expression of miR‐320 was found to be highly down‐regulated in peripheral blood of AD patients and confirmed to be negatively correlated with the expression of MMP, which is produced by monocytes or macrophages.^69^ MMPs, which are enzymes with similar structures but different functions, are classified into five groups: collagenases, gelatinases, stromelysins, matrilysins and membrane type.^70^ Specific types of MMPs can be produced by different cells, whereas activated monocytes and macrophages can produce multiple MMPs.^71^ Physiological remodelling of the ECM and vascular system requires MMP expression; however, its overexpression has adverse effects. For example, MMP‐2 is related to the increased risk of AD.^26^ Collectively, results suggest that miR‐320 can regulate MMP expression post‐transcriptionally. In addition, the overexpression of MMP contributes to the ECM degradation, thereby influencing the progression of AD. However, further studies are required to determine whether miR‐320 can also regulate the function of VSMCs, ECs and other upstream elements.

Hsa‐miR‐320d and hsa‐miR‐582 expression detected by next‐generation sequencing (NGS) and gene expression microarrays had >10‐fold reduction in human AD thoracic aortic fragments. Transfecting miR‐320d mimic can alter the expression of 577 genes, whereas transfection with miR‐582 altered the expression of 203 genes. Of the 20,972 genes listed in the gene ontology database, only 2.7% (miR‐320d) and 1% (miR‐582) had altered expression levels. Interestingly, several genes were discovered to be related to the apoptotic pathway. In particular, *TRIAP1* and *NET1* may be downstream targets of miR‐320d, whereas *COL1A1* and *SPP1*, which are related to extracellular matrix degradation, may be downstream targets of miR‐582.^21^ The apoptotic pathways in VSMCs play an essential role in the development of AD. Das et al confirmed that the enrichment of S100A12, which is a pro‐inflammatory protein, can activate caspase 3 (CASP3) and promote apoptosis in human AD tissues.^72^ In summary, miR‐320d and miR‐582 can regulate the progression of AD by participating in the apoptotic pathway and serve as potential biomarkers for the diagnosis and treatment of different diseases. However, this hypothesis needs further validation in human or mouse models.

#### 4.1.5 microRNA‐144‐3p (miR‐144‐3p)

miR‐144‐3p has increased expression levels in AoSMCs derived from AD patients dissection specimens. Notably, the down‐regulation of miR‐144‐3p expression through adeno‐associated viruses can reduce the incidence and severity of AD. Bioinformatic analysis and dual‐luciferase reporter assay confirmed that miR‐144‐3p binds to the 3′‐UTR of the tropoelastin (TE) mRNA to inhibit protein translation.^73^ TE is a soluble monomer of elastin, a polymeric extracellular protein that determines the ductility and elastic rebound of many cells, accounting for more than 50% of the AoSMC dry weight. The polymerization of TE and other protein components forms the extracellular elastic matrix, which determines the mechanical stability and physical properties of tissues.^74^ Moreover, TE polymorphism defects greatly contribute to the initiation of AD.^75^ In a BAPN‐induced mouse AD model, the inhibition of miR‐144‐3p expression resulted in the reduced incidence of AD from 90% to 50%. This indicates that miR‐144‐3p plays a vital role in the pathogenesis of AD and may serve as a potential therapeutic target.

#### 4.1.6 microRNA‐146b (miR‐146b) and microRNA‐146a‐5p (miR‐146a‐5p)

It was reported that miR‐146b expression was significantly increased in peripheral blood and aortic wall tissues of the TAAD group compared with the control group (*P* < .001). In addition, the differential expression of miR‐146b positively correlated with the high risk of AD. Many ways were used to predict miR‐146b–related target genes, identifying *NF‐κB1*, tumour necrosis factor receptor‐associated factor 6 (*TRAF6*), *MMP16* and actin alpha 2 (*ACTA2*).^76^ It has been previously confirmed that atherosclerosis and autoimmune inflammation are associated with the excessive activation of NF‐κB1.^77^ Therefore, NF‐κB1 may also be involved in the occurrence of AD through the immune‐inflammatory response. miR‐146b can also target the 3′‐UTR of *TRAF6* and promote vascular EC inflammation by regulating the Toll‐like receptor (TLR) immune signalling pathway. Hence, these predicted gene targets may also be involved in the formation of AD.^78^ MMPs, which can degrade the ECM and regulate immune‐inflammatory responses, are associated with apoptosis and inflammatory signalling pathways, and therefore, MMPs play an important role in vascular remodelling.^24^ Mutations in *ACTA2* have been reported to regulate the TGF‐β signalling pathway; thus, miR‐146b can participate in the development of AD by regulating the TGF‐β signalling pathway.^79^ In summary, the increased expression of miR‐146b may be related to the incidence and severity of TAAD. Therefore, miR‐146b may be a potential biomarker that can predict the risk and severity of TAAD rupture and become a basis for selecting appropriate treatment methods.

miR‐146a‐5p was discovered to participate in the regulation of the immune system and myeloid tumorigenesis,^80^ as well as cell proliferation and migration.^81^ *SMAD4*‐mediated regulation resulted in significantly increased miR‐146a‐5p expression (*P* < .05) and elevated VSMC proliferation and migration rates via in Stanford type A AD patients plasma samples and tissue specimens.^82^ However, the small sample size and unknown association between circulating miR‐146a‐5p levels and AD severity limit the potential of miR‐146a‐5p as a biomarker for predicting prognosis.

#### 4.1.7 microRNA‐30a (miR‐30a)

miR‐30a expression was found to be significantly up‐regulated in human AD ascending aorta specimens, consequently reducing the lysyl oxidase (LOX) expression via translation inhibition in the aortic wall.^83^ LOX is an extracellular, copper‐dependent enzyme that cross‐links collagen and elastin. LOX inactivation inhibits the cross‐linking of collagen and elastin, leading to aortic aneurysm (AA) in inbred mottled mice.^84^ In addition, *LOX* knockout or reduced *LOX* expression of in mice, turkeys and rats was associated with AD.^85^ In AngII‐induced AAD and aortic angiography animal model, the rats pre‐treated with agomiR‐30a have a significantly higher probability of developing AD. Collectively, these results provide new insights into the molecular mechanism of AD formation and highlight the potential of miR‐30a as a potential therapeutic target for AD.

#### 4.1.8 microRNA‐143/145 (miR‐143/145) gene cluster

In AngII‐induced AD model, the down‐regulated expression of the miRNA‐143/145 gene cluster and the switch of VSMCs from a contractile to a synthetic phenotype were associated with the P38‐MAPK signalling pathway, and in AD ascending aorta tissues, the expression of phosphorylated p38‐MAPK increased obviously.^52^ Additionally, the down‐regulation of the miR‐143/145 gene cluster during the pathogenesis of AD can promote VSMC phenotypic switching and aortic media degeneration through the TGF‐β1 signalling pathway.^12^ The miR‐143/145 gene cluster, including miR‐143 and miR‐145, is limited to the adult SMC lineages during development.^86^ Notably, AngII regulates the expression of miR143/145 and promotes the transition of murine VSMCs from a synthetic to a contractile phenotype during the formation of AD.^87^ It was reported that AngII has elevated levels in the serum of AD patients and the overexpression of AngII can induce aortic atherosclerosis and AA by modulating the proliferation, differentiation, apoptosis and hypertrophy of VSMCs.^88^ Recently, it was confirmed that AngII can also activate MAPKs, including signal‐regulated kinase (ERK1/2), JNK and p38, by activating the signalling cascades specifically related to the proliferation, migration, differentiation and fibrosis of VSMC.^89^ However, further studies are needed to verify whether the down‐regulation of the miR‐143/145 gene cluster activates p38 signals before or after the onset of AD.

#### 4.1.9 microRNA‐4787‐5p (miR‐4787‐5p) and microRNA‐4306 (miR‐4306)

miRNA microarray analysis revealed that the expression levels of circulating miR‐4787‐5p and miR‐4306 were >2‐fold higher in AAD patients.^90^ Furthermore, the combination value of miR‐4787‐5p and miR‐4306 was equal or greater than D‐dimer during the early diagnosis of AAD. Bioinformatic analysis predicted *PKD1* and *TGF‐β1* as potential targets of miR‐4787‐5p and miR‐4306, respectively. The results of dual‐luciferase reporter assay validated this prediction. *PKD1* and *TGF‐β1* were suggested to be associated with the development of aortic diseases, and these two were observed to have decreased expression levels in AAD patients.^90^, ^91^ In summary, miR‐4787‐5p and miR‐4306 may play important roles in the early diagnosis of AAD. However, more samples need to be studied to verify the diagnostic value of miR‐4787‐5p and miR‐4306 as potential biomarkers in AAD.

#### 4.1.10 microRNA‐26b (miR‐26b)

High‐mobility group AT‐hook 2 (*HMGA2*) plays an essential role in cellular proliferation and differentiation during embryonic development and is involved in the development of cardiovascular diseases, including atherosclerosis and cardiac lipomas.^92^ Furthermore, *HMGA2* overexpression in AAD occurs in a let‐7d–independent manner, which influences AAD through epithelial‐mesenchymal transition (EMT).^93^ It was confirmed that the miR‐26b/*HMGA2* axis contributes to TAAD development through the TGF‐β signalling pathway. miR‐26b regulates the expression of *HMGA2*, thereby indirectly regulating VSMC proliferation and apoptosis, and activates the TGF‐β/SMAD3 signalling pathway to promote VSMC proliferation.^94^ miR‐26b was found to be negatively correlated with the risk and severity of TAAD, and miR‐26b expression was decreased in ascending aorta tissue of TAAD patients. It was confirmed as a biomarker for TAAD diagnosis, which can predict the risk of AD, assess the prognosis and serve as a basis for choosing the timing of surgery.^44^

## 5 LONG NON‐CODING RNAS (LNCRNAS) IN AORTIC DISSECTION

Long non‐coding RNAs (lncRNAs), generally 200 nt long, are reported to be three‐dimensional (3D) regulators of transcription and translation by acting as molecular decoys and scaffolds or by binding the guide ribonucleoprotein complexes to their targets. The cellular expression of lncRNAs is tissue‐specific; however, some are only expressed at specific stages of eukaryotic development. Moreover, these can be host genes for the transcription of miRNAs in the nucleus or act as miRNA sponges in the cytoplasm.^95^ In recent years, lncRNAs were found to play an important role in the occurrence and development of cardiovascular diseases.^96^ Most lncRNAs have significant spatiotemporal expression and specificity during tissue differentiation and development, making them better biomarkers for the diagnosis of AD (Figure 2).

High‐throughput sequencing (HTS) technology was previously used to study the expression profile of lncRNAs in human TAD tissues. Several genes were found to be differentially expressed in human TAD, including 269 lncRNAs (110 down‐regulated and 159 up‐regulated) and 2255 mRNAs (961 down‐regulated and 1,294 up‐regulated). Results also demonstrated the positive correlations between lncRNAs (ENSG00000269936, lncRNA‐1421) and their adjacent mRNAs (*MAP2K6*, *FBLN5*, *ACTA2*, *TIMP3*). The ENSG00000248508, ENSG00000226530 and EG00000259719 lncRNAs and their upstream target, *RUNX1*, were also up‐regulated.^97^ Previous studies have reported that *RUNX1* can regulate *MMP9* expression and their increased expression contributes to the immune response in abdominal AA.^98^ Therefore, the identified lncRNAs that are regulated by *RUNX1* may have important functions in the development of TAD.

### 5.1 lncRNA‐XIST, ENSG00000269936 and lncRNA‐1421

The lncRNA‐miRNA‐mRNA network revealed that lncRNA‐XIST directly binds to miR‐17‐5p to regulate *p21* expression. lncRNA‐XIST and *p21* were up‐regulated, whereas has‐miR‐17‐5p was down‐regulated in TAD tissues. miR‐17‐5p can regulate the expression of *p21*, *PKD2* and *SOD2*, which are all involved in the regulation of aortic diseases.^99^, ^100^, ^101^ Therefore, lncRNA‐XIST can indirectly regulate the expression of *p21* through sponging miR‐17‐5p and ultimately participate in the development of TAD. In addition, ENSG00000269936 mediates AD development by cis‐targeting its adjacent gene, *MAP2K6*, a member of the MAPK signalling pathway. Notably, the p38 MAPK signalling pathway was reported to regulate the factors associated with AD progression.^102^ lncRNA‐1421 regulates TAD development by trans‐regulating the expression of *TIMP3*, *FBLN5* and *ACTA2*, which participate in AD development through different ways.^103^, ^104^, ^105^ Collectively, these studies provide new insights into the molecular mechanisms of AD and may reveal the important function of lncRNAs in creating future prevention strategies.

### 5.2 PTENP1

The lncRNA PTENP1 is a pseudogene of the tumour suppressor gene phosphatase and tensin homologue deleted on chromosome ten (PTEN).^106^ PTENP1 and PTEN have an endogenous competitive relationship. PTENP1 can act as a molecular sponge of miR‐21 to bind with miR‐21, release the expression of PTEN, further inhibit downstream threonine kinase (Akt) signal transduction and finally reduce the expression of cyclin D1 and cyclin E. Increased *PTEN* expression further promotes apoptosis and inhibits the proliferation of human aortic smooth muscle cells (HASMCs). In contrast, PTENP1 silencing inhibits the H_2_O_2_‐induced HASMC apoptosis. In AngII‐induced mouse AA model, PTENP1 overexpression enhanced the apoptosis of AoSMCs and aggravated the formation of aneurysms. Therefore, PTENP1 plays an important role in maintaining HASMC homeostasis and may serve as a potential target for therapeutic intervention of AD or AA.^107^

In TAD, a total of 765 lncRNAs and 619 mRNAs have been identified to be aberrantly expressed. Further, 16 lncRNAs and their target genes may be associated with TAD pathogenesis. The qRT‐PCR results showed that the expression levels of five lncRNAs (RP11‐536K7.5, AX746823, lncP2RX7, HIF1A‐AS2 and RP11‐69I8.3) and 6 mRNAs (*CTGF*, *IL2RA*, *HIF‐1A*, *P2RX7*, *CDKN2B* and *RUNX1*) significantly increased in TAD, suggesting that these are potentially involved in TAD occurrence and development. In addition, RP11‐69I8.3, AX746823, RP11‐536K7.5 and lncP2RX7 were discovered to be related to the activation of several protein‐coding genes involved with connective tissue development (*CTGF*), nuclear transcription (*RUNX1*), inflammation (*IL2RA*) and nuclear receptors (*P2RX7*). Previous studies demonstrated that *HIF‐1A*, *CTGF*, *CDKN2B*, *P2X7*, *RUNX1* and *IL2RA* can regulate specific factors that are involved in the formation and development of AD.^98^, ^108^, ^109^, ^110^, ^111^, ^112^ However, further studies are needed to determine whether these genes are also TAD‐related and what cell types the proteins are expressed in Ref.^113^

## 6 CIRCULAR RNAS (CIRCRNAS) IN AORTIC DISSECTION

circRNAs are a special group of novel endogenous lncRNAs that can bind to and interact with miRNAs and play an important regulatory role at the transcriptional or post‐transcriptional level.^114^ The circRNA expression profiles are usually analysed by high‐throughput RNA sequencing (RNA‐Seq) technology and computational analysis, revealing numerous circRNAs with differential expression.^115^ Unlike miRNAs and lncRNAs, circRNAs have closed‐ring structures and therefore have no 5′–3′ polarity and polyA tail; however, circRNAs do retain the sequence direction according to their mRNA of origin. circRNAs are more stable than linear RNAs and not easily degraded by RNA exonuclease or RNase R, and thus, the expression of circRNA in various human cells and tissues is detectable. The biological functions of circRNAs include the following: (a) sponge adsorbents of miRNAs as competitors of endogenous mRNAs; (b) regulate variable shear or transcriptional processes; and (b) regulate the expression of parental genes.^116^ These characteristics determine the important functions of circRNAs in transcription and post‐transcription, suggesting that these may be ideal biomarkers for disease diagnosis (Figure 2).

### 6.1 circRNA‐101238

Zou et al found 8,173 circRNAs that are differentially expressed in human type A TAD. The qRT‐PCR results showed that hsa‐circRNA‐101238, hsa‐circRNA‐002271, hsa‐circRNA‐104634, hsa‐circRNA‐104349, hsa‐circRNA‐102771, COL1A1 and COL6A3 were up‐regulated, whereas FLNA, hsa‐circRNA‐102683, hsa‐circRNA‐103458 and hsa‐circRNA‐005525 were down‐regulated. Some TAD‐associated miRNAs can be regulated by several circRNAs. For example, miRNA expression profile analysis revealed that the expression of hsa‐miR‐320a, hsa‐miR‐320b and hsa‐miR‐320c was inhibited by hsa‐circRNA‐101238 in TAD aortic specimens.^85^ Therefore, the higher the expression level of circRNA‐101238 in human TAD tissues, the lower the expression level of the downstream target miR‐320a, leading to increased *MMP9* expression. SMC phenotypic transformation or apoptosis was promoted by the up‐regulation of hsa‐circRNA‐104349 and hsa‐circRNA‐104634, which interacted with hsa‐miR‐26a‐3p and hsa‐miR‐145‐3p, respectively.^117^ hsa‐circRNA‐102683 and hsa‐circRNA‐104033 can inhibit the expression of hsa‐miR‐29b‐1‐5p and hsa‐miR‐195‐3p, respectively, thereby promoting apoptosis and ECM degradation in the aortic wall, as well as stimulating collagen remodelling.^118^ Thus, the identified differentially expressed circRNAs may function in the development of TAD through multiple biological processes.^119^

### 6.2 circ‐MARK3

Tian et al also performed RNA‐Seq on human acute Stanford type A aortic dissection (AAAD) diseased ascending aortic specimens and discovered that 506 circRNAs were significantly differentially expressed.^47^ The top 10 highly differentially expressed circRNAs, with 2‐ to 5‐fold change in expression, included the up‐regulated circARHGAP26, circUBA2, circCHSY1, circIQGAP1, circMBNL1, circMED13, circRAB7A and circMYH10 and the down‐regulated circCEP70 and circFAM120B. The circRNA‐miRNA‐mRNA network analysis results suggested that circMARK3 could regulate the expression of Fgr, a tyrosine‐protein kinase. Fgr participates in cellular signal transmission and regulates the immune response and release of inflammatory factors.^120^ Additionally, the diagnostic value of serum circMARK3 was further validated by ROC analysis, revealing 0.9344 AUC, 90.0% sensitivity and 86.7% specificity. The RNAhybrid programme predicted the miR‐1273g‐3p‐circRNA interaction pair, suggesting that the combination of serum miR‐1273‐3p and circMARK3 can greatly improve their diagnostic efficiency as biomarkers of AD. In summary, the interaction of circMARK3‐miR‐1273‐Fgr was believed to have certain clinical significance and combining circRNAs with other biomarkers can provide higher diagnostic value. However, the direct interaction between circMARK3‐miR‐1273‐Fgr needs to be verified by further biological experiments.

## 7 DISCUSSION

Previous studies have identified many ncRNAs, such as miRNAs, lncRNAs and circRNAs, which are involved in the pathogenesis of AD. Several miRNAs with therapeutic potential, such as miR‐21, miR‐134‐5p, miR‐144‐3p and miR‐30a, have been reported in animal AD models and cross‐validated in human AD tissue samples. In contrast, although several lncRNAs have been implicated in certain pathophysiological processes during AD development, only lncRNAs‐XIST, PTENP1, lncP2RX7 and ENSG00000269936 have been validated. In addition, circ‐101238 and circ‐MARK3 may have potential diagnostic value. As mentioned above, some miRNAs that are involved in the development of AD have already been studied using animal models. Currently, scientific research is more advanced and clinical applications are continuously being developed; these will complement the in‐depth research on the potential applications of miRNAs in clinical settings. Likewise, numerous lncRNAs and circRNAs that can potentially regulate AD have been detected through sequencing and microarray experiments. However, the exact functions and mechanisms of these ncRNAs are unknown and require further investigations.

To understand the biological and pathophysiological mechanisms associated with AD occurrence and progression, the abnormally expressed miRNAs, lncRNAs and circRNAs must be identified and validated in related animal models or human AD tissues. Extracting the RNA from key cell types, such as ECs, SMCs and immune cells, will help obtain specific insights into the cell‐type specific changes during AD development. After verifying their differential expression, the molecular mechanisms through which these ncRNAs regulate AD development must also be tested in vivo and in vitro.

In most cases, AD has very poor prognosis; however, the development of interventional therapy has markedly improved the current therapeutic efficacy. Recently, a new method for AD treatment previously used for the nucleic acid therapy of thoracic aortic dissection (TAD) through a multifunctional cationic nanoparticle system has been reported.^121^ This system has no therapeutic effect on its own and is only a microRNA (miRNA) carrier that can be stored in the blood and will not damage the organs. This treatment strategy is suitable for people with TAD susceptibility, such as patients with Marfan syndrome. However, this delivery system has low specificity because its target is exposed to the pathological tissue of type IV collagen. Currently, an effective treatment for AD is still unavailable. Therefore, in‐depth studies focusing on the pathogenesis, new interventions and treatment strategies are required.

The rapid growth of miRNA research provides new insights for future studies on the identified potential biomarkers and therapeutic targets. However, some research gaps still need to be addressed. First, ncRNA biology research focuses on the effects of miRNAs, lncRNAs or circRNAs on VSMCs. This needs to be extended to the effect of miRNAs or lncRNAs on other cellular components, such as ECs, fibroblasts and macrophages. Second, besides miRNAs, lncRNAs and circRNAs, other ncRNAs such as repeat‐associated small interfering RNAs (rasiRNAs) and Piwi‐interacting RNAs (piRNAs) may also be involved in the development of cardiovascular pathology, and thus, these need to be investigated. Third, additional studies on miRNA medicinal chemistry and delivery methods are needed to create strategies for the local, systemic and safe delivery of ncRNA‐based therapeutics, as well as subsequent research in clinically relevant animal models and corresponding in vivo imaging systems.

In conclusion, in‐depth research of ncRNAs will provide potential therapeutic targets and biomarkers to monitor the progress and severity of AD in patients. Early and correct diagnosis of AD can significantly reduce mortality in human patients. Therefore, further efforts to identify other candidate biomarkers and to elucidate their underlying mechanisms, as well as their diagnostic and therapeutic potentials, are required.

## CONFLICT OF INTERESTS

None.

## AUTHOR CONTRIBUTIONS

**Mengdie Cheng:** Data curation (lead); formal analysis (lead); resources (lead); writing‐original draft (lead). **Yanyan Yang:** Conceptualization (equal); funding acquisition (equal); resources (equal); writing‐review and editing (equal). **Hai Xin:** Conceptualization (supporting); resources (supporting). **Tingyu Zong:** Data curation (supporting). **Xingqiang He:** Data curation (supporting); resources (supporting). **Min Li:** Formal analysis (supporting); validation (supporting). **Tao Yu:** Conceptualization (lead); funding acquisition (equal); project administration (lead); supervision (equal); validation (lead); visualization (lead); writing‐review and editing (lead). **Hui Xin:** Funding acquisition (equal); project administration (equal); supervision (equal).
